# An Ossified Man Broken

**Published:** 1891-12

**Authors:** 


### An Ossified Man Broken.

As ossified man, who had been exhibited in many places around the country, was being carried up-stairs in his home in this city the other evening when the cot was tipped up a little and he rolled off down the steps. When picked up it was found that he had fractures of the right thigh, left leg and index finger of the right hand.

